# Supplementary material for: HIVprotI: an integrated web based platform for prediction and design of HIV proteins inhibitors
Source: J Cheminform. 2018 Mar 9;10:12. doi: 10.1186/s13321-018-0266-y (PMC5845081; doi:10.1186/s13321-018-0266-y)
Supplement: Supplementary file 2 — Additional file 2. Source code of HIVProtI web server. [file 13321_2018_266_MOESM2_ESM.zip › HIVprotI_Source-code/hivprotI/weka-3-6-6/doc/weka/filters/supervised/attribute/AddClassification.html]

AddClassification


---


|  |  |  |
| --- | --- | --- |
| **Overview** \| **Package** \| **Class** \| **Tree** \| **Deprecated** \| **Index** \| **Help** \| **Weka's home** | |  |
| PREV CLASS   **NEXT CLASS** | **FRAMES**    **NO FRAMES**     **All Classes** |  |
| SUMMARY: NESTED \| FIELD \| CONSTR \| METHOD | DETAIL: FIELD \| CONSTR \| METHOD |  |


---


## weka.filters.supervised.attribute Class AddClassification

```
java.lang.Object
  weka.filters.Filter
      weka.filters.SimpleFilter
          weka.filters.SimpleBatchFilter
              weka.filters.supervised.attribute.AddClassification
```

**All Implemented Interfaces:**: java.io.Serializable, CapabilitiesHandler, OptionHandler, RevisionHandler

---

``` public class AddClassification extends SimpleBatchFilter ```

A filter for adding the classification, the class distribution and an error flag to a dataset with a classifier. The classifier is either trained on the data itself or provided as serialized model.


Valid options are:

```
 -D
  Turns on output of debugging information.
```

```
 -W <classifier specification>
  Full class name of classifier to use, followed
  by scheme options. eg:
   "weka.classifiers.bayes.NaiveBayes -D"
  (default: weka.classifiers.rules.ZeroR)
```

```
 -serialized <file>
  Instead of training a classifier on the data, one can also provide
  a serialized model and use that for tagging the data.
```

```
 -classification
  Adds an attribute with the actual classification.
  (default: off)
```

```
 -remove-old-class
  Removes the old class attribute.
  (default: off)
```

```
 -distribution
  Adds attributes with the distribution for all classes
  (for numeric classes this will be identical to the attribute
  output with '-classification').
  (default: off)
```

```
 -error
  Adds an attribute indicating whether the classifier output
  a wrong classification (for numeric classes this is the numeric
  difference).
  (default: off)
```

**Version:**
:   $Revision: 6900 $

**Author:**
:   fracpete (fracpete at waikato dot ac dot nz)

**See Also:**: Serialized Form

---

**Constructor Summary**

`AddClassification()`


| **Method Summary** | |
| --- | --- |
| `java.lang.String` | `classifierTipText()`             Returns the tip text for this property. |
| `Capabilities` | `getCapabilities()`             Returns the Capabilities of this filter. |
| `Classifier` | `getClassifier()`             Gets the classifier used by the filter. |
| `java.lang.String[]` | `getOptions()`             Gets the current settings of the classifier. |
| `boolean` | `getOutputClassification()`             Get whether the classifiction of the classifier is output. |
| `boolean` | `getOutputDistribution()`             Get whether the classifiction of the classifier is output. |
| `boolean` | `getOutputErrorFlag()`             Get whether the classifiction of the classifier is output. |
| `boolean` | `getRemoveOldClass()`             Get whether the old class attribute is removed. |
| `java.lang.String` | `getRevision()`             Returns the revision string. |
| `java.io.File` | `getSerializedClassifierFile()`             Gets the file pointing to a serialized, trained classifier. |
| `java.lang.String` | `globalInfo()`             Returns a string describing this filter. |
| `java.util.Enumeration` | `listOptions()`             Returns an enumeration describing the available options. |
| `static void` | `main(java.lang.String[] args)`             runs the filter with the given arguments. |
| `java.lang.String` | `outputClassificationTipText()`             Returns the tip text for this property. |
| `java.lang.String` | `outputDistributionTipText()`             Returns the tip text for this property. |
| `java.lang.String` | `outputErrorFlagTipText()`             Returns the tip text for this property. |
| `java.lang.String` | `removeOldClassTipText()`             Returns the tip text for this property. |
| `java.lang.String` | `serializedClassifierFileTipText()`             Returns the tip text for this property. |
| `void` | `setClassifier(Classifier value)`             Sets the classifier to classify instances with. |
| `void` | `setOptions(java.lang.String[] options)`             Parses the options for this object. |
| `void` | `setOutputClassification(boolean value)`             Set whether the classification of the classifier is output. |
| `void` | `setOutputDistribution(boolean value)`             Set whether the Distribution of the classifier is output. |
| `void` | `setOutputErrorFlag(boolean value)`             Set whether the classification of the classifier is output. |
| `void` | `setRemoveOldClass(boolean value)`             Set whether the old class attribute is removed. |
| `void` | `setSerializedClassifierFile(java.io.File value)`             Sets the file pointing to a serialized, trained classifier. |

**Methods inherited from class weka.filters.SimpleBatchFilter**

`batchFinished, input`

**Methods inherited from class weka.filters.SimpleFilter**

`debugTipText, getDebug, setDebug, setInputFormat`

**Methods inherited from class weka.filters.Filter**

`batchFilterFile, filterFile, getCapabilities, getOutputFormat, isFirstBatchDone, isNewBatch, isOutputFormatDefined, makeCopies, makeCopy, numPendingOutput, output, outputPeek, toString, useFilter, wekaStaticWrapper`

**Methods inherited from class java.lang.Object**

`equals, getClass, hashCode, notify, notifyAll, wait, wait, wait`

**Constructor Detail**

### AddClassification

```
public AddClassification()
```


**Method Detail**

### globalInfo

```
public java.lang.String globalInfo()
```

:   Returns a string describing this filter.

    :   **Specified by:**: `globalInfo` in class `SimpleFilter`
    :   **Returns:**: a description of the filter suitable for displaying in the explorer/experimenter gui

---


### listOptions

```
public java.util.Enumeration listOptions()
```

:   Returns an enumeration describing the available options.

    :   **Specified by:**: `listOptions` in interface `OptionHandler` **Overrides:**: `listOptions` in class `SimpleFilter`
    :   **Returns:**: an enumeration of all the available options.

---


### setOptions

```
public void setOptions(java.lang.String[] options)
                throws java.lang.Exception
```

:   Parses the options for this object.


    Valid options are:

    ```
     -D
      Turns on output of debugging information.
    ```

    ```
     -W <classifier specification>
      Full class name of classifier to use, followed
      by scheme options. eg:
       "weka.classifiers.bayes.NaiveBayes -D"
      (default: weka.classifiers.rules.ZeroR)
    ```

    ```
     -serialized <file>
      Instead of training a classifier on the data, one can also provide
      a serialized model and use that for tagging the data.
    ```

    ```
     -classification
      Adds an attribute with the actual classification.
      (default: off)
    ```

    ```
     -remove-old-class
      Removes the old class attribute.
      (default: off)
    ```

    ```
     -distribution
      Adds attributes with the distribution for all classes
      (for numeric classes this will be identical to the attribute
      output with '-classification').
      (default: off)
    ```

    ```
     -error
      Adds an attribute indicating whether the classifier output
      a wrong classification (for numeric classes this is the numeric
      difference).
      (default: off)
    ```

    :   **Specified by:**: `setOptions` in interface `OptionHandler` **Overrides:**: `setOptions` in class `SimpleFilter`
    :   **Parameters:**: `options` - the options to use **Throws:**: `java.lang.Exception` - if setting of options fails **See Also:**: `SimpleFilter.reset()`

---


### getOptions

```
public java.lang.String[] getOptions()
```

:   Gets the current settings of the classifier.

    :   **Specified by:**: `getOptions` in interface `OptionHandler` **Overrides:**: `getOptions` in class `SimpleFilter`
    :   **Returns:**: an array of strings suitable for passing to setOptions

---


### getCapabilities

```
public Capabilities getCapabilities()
```

:   Returns the Capabilities of this filter.

    :   **Specified by:**: `getCapabilities` in interface `CapabilitiesHandler` **Overrides:**: `getCapabilities` in class `Filter`
    :   **Returns:**: the capabilities of this object **See Also:**: `Capabilities`

---


### classifierTipText

```
public java.lang.String classifierTipText()
```

:   Returns the tip text for this property.

    :   **Returns:**: tip text for this property suitable for displaying in the explorer/experimenter gui

---


### setClassifier

```
public void setClassifier(Classifier value)
```

:   Sets the classifier to classify instances with.

    :   **Parameters:**: `value` - The classifier to be used (with its options set).

---


### getClassifier

```
public Classifier getClassifier()
```

:   Gets the classifier used by the filter.

    :   **Returns:**: The classifier to be used.

---


### serializedClassifierFileTipText

```
public java.lang.String serializedClassifierFileTipText()
```

:   Returns the tip text for this property.

    :   **Returns:**: tip text for this property suitable for displaying in the explorer/experimenter gui

---


### getSerializedClassifierFile

```
public java.io.File getSerializedClassifierFile()
```

:   Gets the file pointing to a serialized, trained classifier. If it is
    null or pointing to a directory it will not be used.

    :   **Returns:**: the file the serialized, trained classifier is located in

---


### setSerializedClassifierFile

```
public void setSerializedClassifierFile(java.io.File value)
```

:   Sets the file pointing to a serialized, trained classifier. If the
    argument is null, doesn't exist or pointing to a directory, then the
    value is ignored.

    :   **Parameters:**: `value` - the file pointing to the serialized, trained classifier

---


### outputClassificationTipText

```
public java.lang.String outputClassificationTipText()
```

:   Returns the tip text for this property.

    :   **Returns:**: tip text for this property suitable for displaying in the explorer/experimenter gui

---


### getOutputClassification

```
public boolean getOutputClassification()
```

:   Get whether the classifiction of the classifier is output.

    :   **Returns:**: true if the classification of the classifier is output.

---


### setOutputClassification

```
public void setOutputClassification(boolean value)
```

:   Set whether the classification of the classifier is output.

    :   **Parameters:**: `value` - whether the classification of the classifier is output.

---


### removeOldClassTipText

```
public java.lang.String removeOldClassTipText()
```

:   Returns the tip text for this property.

    :   **Returns:**: tip text for this property suitable for displaying in the explorer/experimenter gui

---


### getRemoveOldClass

```
public boolean getRemoveOldClass()
```

:   Get whether the old class attribute is removed.

    :   **Returns:**: true if the old class attribute is removed.

---


### setRemoveOldClass

```
public void setRemoveOldClass(boolean value)
```

:   Set whether the old class attribute is removed.

    :   **Parameters:**: `value` - whether the old class attribute is removed.

---


### outputDistributionTipText

```
public java.lang.String outputDistributionTipText()
```

:   Returns the tip text for this property.

    :   **Returns:**: tip text for this property suitable for displaying in the explorer/experimenter gui

---


### getOutputDistribution

```
public boolean getOutputDistribution()
```

:   Get whether the classifiction of the classifier is output.

    :   **Returns:**: true if the distribution of the classifier is output.

---


### setOutputDistribution

```
public void setOutputDistribution(boolean value)
```

:   Set whether the Distribution of the classifier is output.

    :   **Parameters:**: `value` - whether the distribution of the classifier is output.

---


### outputErrorFlagTipText

```
public java.lang.String outputErrorFlagTipText()
```

:   Returns the tip text for this property.

    :   **Returns:**: tip text for this property suitable for displaying in the explorer/experimenter gui

---


### getOutputErrorFlag

```
public boolean getOutputErrorFlag()
```

:   Get whether the classifiction of the classifier is output.

    :   **Returns:**: true if the classification of the classifier is output.

---


### setOutputErrorFlag

```
public void setOutputErrorFlag(boolean value)
```

:   Set whether the classification of the classifier is output.

    :   **Parameters:**: `value` - whether the classification of the classifier is output.

---


### getRevision

```
public java.lang.String getRevision()
```

:   Returns the revision string.

    :   **Specified by:**: `getRevision` in interface `RevisionHandler` **Overrides:**: `getRevision` in class `Filter`
    :   **Returns:**: the revision

---


### main

```
public static void main(java.lang.String[] args)
```

:   runs the filter with the given arguments.

    :   **Parameters:**: `args` - the commandline arguments


---


|  |  |  |
| --- | --- | --- |
| **Overview** \| **Package** \| **Class** \| **Tree** \| **Deprecated** \| **Index** \| **Help** \| **Weka's home** | |  |
| PREV CLASS   **NEXT CLASS** | **FRAMES**    **NO FRAMES**     **All Classes** |  |
| SUMMARY: NESTED \| FIELD \| CONSTR \| METHOD | DETAIL: FIELD \| CONSTR \| METHOD |  |


---
